# Buruli ulcer in a nine-month-old boy

**DOI:** 10.1308/003588412X13373405385692

**Published:** 2012-10

**Authors:** S Tsukagoshi, TCB Dehn

**Affiliations:** ^1^Oxford Deanery,UK; ^2^Royal Berkshire NHS Foundation Trust,UK

**Keywords:** Tropical skin ulceration, Buruli ulcer, Mycobacterium ulcerans, Surgery

## Abstract

The diagnosis of Buruli ulcer should be considered in all painless undermined ulcers in the tropics. The diagnosis and treatment are a challenge in rural settings despite the well established tuberculosis programmes. Immediate commencement on rifampicin and streptomycin is essential to halt the progression of disease and to, hopefully, reverse it. Surgery is indicated in those with complex ulcers or with complications. We report the case of a nine-month-old boy presenting to visiting British surgeons in a district hospital in Uganda with multiple ulcers to the right forearm.

## Case history

**Figure 1:**
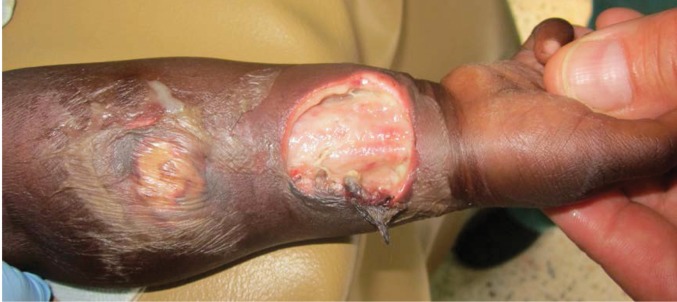
Volar lesions on day 3 showing undermined edges and slough at the base

A nine-month-old male baby presented to Kamuli Mission Hospital, a rural Ugandan hospital, with right forearm oedema and lethargy. He was commenced on gentamicin, metronidazole and ceftriaxone by local clinical officers. Three days after admission, he developed two blisters to the volar aspect of the wrist and forearm (Fig 1). Three days later, these blisters had developed into two painless undermined ulcers revealing underlying flexor muscles, with a third lesion appearing on the dorsum of the wrist.

**Figure 2:**
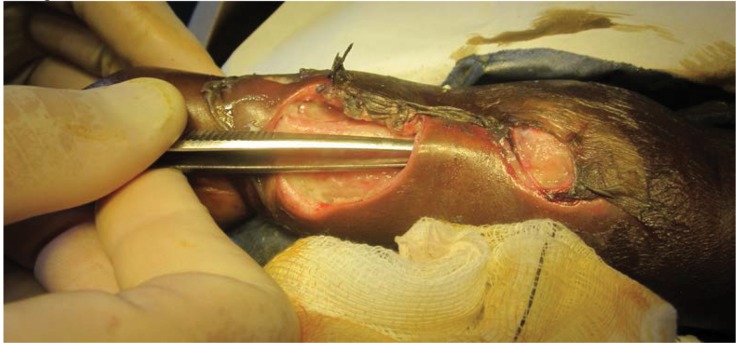
Subcutaneous communication between lesions

**Figure 3:**
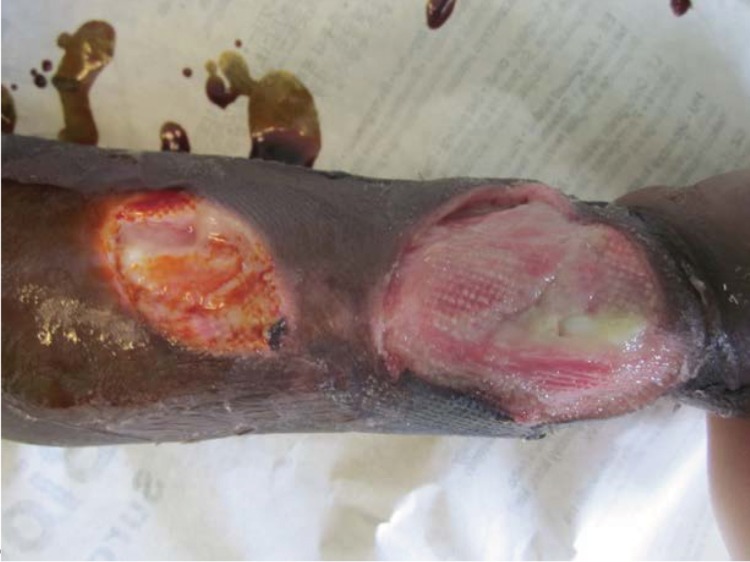
Wounds on day 10, now healthy. Note the exposed wrist joint capsule.

These were washed out and debrided under intravenous ketamine anaesthesia. The ulcers communicated subcutaneously (Fig 2). The flexor tendons were visible, the extensor tendons were destroyed and the wrist joint capsule was exposed (Fig 3). The wounds were dressed with a non-adhesive dressing and the hand was elevated in a sling. The patient was commenced on a combined rifampicin/isoniazid tablet at a dose of rifampicin 10mg/kg since no other preparations were available (including streptomycin). All other antibiotics were discontinued.

On day 7, the patient was taken to theatre for a further washout and dressing change. Ziehl–Neelsen and Gram stains of pus and debrided tissue were reported as negative. Further wound debridements were undertaken on day 8 and 10, by which time the ulcers had improved in appearance and the patient had recommenced normal feeding.

Following family and financial discussions, the patient was transferred to a plastic surgery unit in Kampala. Additionally, he was diagnosed with osteomyelitis of the distal radius and was due to undergo a sequestrectomy before skin grafting. All three ulcers had reduced in size by 50% with conservative therapy.

## Discussion

Buruli ulcers are aggressively destructive skin lesions caused by *Mycobacterium ulcerans*.^1^ First described by Sir Albert Cook in 1897, large numbers of cases were reported in the Buruli district of Uganda (now known as Nakasongola). The peak incidence occurs in those aged 5–15 years but it can occur at any age.^1,2^ The exact mode of transmission is unknown. However, anecdotal evidence suggests that it is an environmental pathogen in contaminated water.^1^ An increase in cases reported in a Rwandan refugee population settling near the Nile in the 1960s and 1970s further confirmed its waterborne transmission. Recent studies have isolated the *Mycobacterium* in the salivary glands of aquatic insects.^1,2^ The disease has been reported in over 30 tropical and subtropical countries across Africa, Asia, the Americas and Australia.^2^

The initial lesion can vary from a small, firm, painless, subcutaneous nodule, a firm plaque or an oedematous lesion.^1^ These lesions break down, forming characteristic painless ulcers with undermined edges.^1,3^ Nearly all occur on the limbs.^1–3^ *M ulcerans* produces mycolactone toxin, which is responsible for the destruction of lipocytes and fibroblasts in subcutaneous tissue, leading to the ulceration and communication between lesions.^1^

The oedematous type is the most aggressive form where swelling and ulceration can occur within days. Diagnosis is principally clinical, especially with the limited resources in endemic countries. A third of nodules may heal spontaneously.^1^ Although the overall mortality is low, extensive disease can lead to significant morbidity.^2^ Culture, histopathology and polymerase chain reaction are usually unavailable. The Ziehl–Neelsen stain has only 40–43% sensitivity.^1,2^

In the reported case, the cardinal feature suggestive of Buruli ulcer was the rapid onset oedematous lesion that broke down to form multiple painless undermined ulcers with subcutaneous communications. These were responsive to rifampicin. Ziehl–Neelsen staining was negative but this does not exclude the diagnosis.^4^

Previously, the mainstay of management was excision with a wide margin due to the presence of mycobacteria in the surrounding tissue.^1^ Recurrence rates following surgery alone lie between 16% and 30%.^2^ Unfortunately, surgery is often inaccessible and many cases are left untreated with huge morbidity outcomes.

The World Health Organization has categorised these ulcers according to size with advice on appropriate management.^2^ All are to be treated with antibiotics. Those greater than 15cm, including multiple ulcers or those with osteomyelitis, should be treated with antibiotics and surgery. An eight-week course of combination therapy of rifampicin (10mg/kg) and streptomycin (15mg/kg) is recommended to reduce the risk of resistance. Antibiotics eliminate the mycobacteria from the tissue, remove and also reduce the size of the lesion, and diminish the need for extensive surgery. Excision, skin grafting and amputation are the most common procedures required. Post-operative recurrence has been reduced to less than 2% with pre-operative antibiotics.

The differential diagnoses include tropical pyomyositis, which is a primary bacterial skeletal muscle infection with abscess formation commonly affecting the hip and thigh.^4^ However, in this case, the lesion was painless throughout and ulceration is not characteristic of this disease. Pyomyositis is less rapid in onset and the patient did not respond to the antistaphylococcal antibiotics. Gram staining of the collected pus was negative and it was therefore felt that this was an unlikely cause to his symptoms.

Due to the limited knowledge of its transmission, there are no public health measures to control the spread.^1^ Currently, no vaccine is available. Education of local populations has facilitated earlier diagnosis and therefore earlier treatment.^2^

## Conclusions

The diagnosis of Buruli ulcer should be considered in all painless undermined ulcers in the tropics. The diagnosis and treatment are a challenge in rural settings despite the well established tuberculosis programmes. Immediate commencement on rifampicin and streptomycin is essential to halt the progression of disease and to, hopefully, reverse it. Surgery is indicated in those with complex ulcers or with complications.
